# Prediction of Antibacterial Activity from Physicochemical Properties of Antimicrobial Peptides

**DOI:** 10.1371/journal.pone.0028549

**Published:** 2011-12-14

**Authors:** Manuel N. Melo, Rafael Ferre, Lídia Feliu, Eduard Bardají, Marta Planas, Miguel A. R. B. Castanho

**Affiliations:** 1 Institute of Molecular Medicine, University of Lisbon, Lisbon, Portugal; 2 Groningen Biomolecular Sciences and Biotechnology Institute, University of Groningen, Groningen, The Netherlands; 3 Laboratori d'Innovació en Processos i Productes de Síntesi Orgànica, Department of Chemistry, University of Girona, Girona, Spain; Aligarh Muslim University, India

## Abstract

Consensus is gathering that antimicrobial peptides that exert their antibacterial action at the membrane level must reach a local concentration threshold to become active. Studies of peptide interaction with model membranes do identify such disruptive thresholds but demonstrations of the possible correlation of these with the in vivo onset of activity have only recently been proposed. In addition, such thresholds observed in model membranes occur at local peptide concentrations close to full membrane coverage. In this work we fully develop an interaction model of antimicrobial peptides with biological membranes; by exploring the consequences of the underlying partition formalism we arrive at a relationship that provides antibacterial activity prediction from two biophysical parameters: the affinity of the peptide to the membrane and the critical bound peptide to lipid ratio. A straightforward and robust method to implement this relationship, with potential application to high-throughput screening approaches, is presented and tested. In addition, disruptive thresholds in model membranes and the onset of antibacterial peptide activity are shown to occur over the same range of locally bound peptide concentrations (10 to 100 mM), which conciliates the two types of observations.

## Introduction

Antimicrobial peptides (AMPs) constitute a broadly defined class of short, cationic peptides produced by virtually all organisms. Since their discovery microbiological methodologies have been employed to characterize their antibacterial action [1], [2]. In turn, the relative simplicity in sequence and secondary structure of AMPs, together with mechanisms that depend largely on membrane interaction [3], made biophysical methodologies the tools of choice to describe the molecular level action of AMPs. A gap, however, separates the two distinct approaches: information from biological studies is seldom correlated to the findings on peptide behavior at the molecular level.

Threshold behavior is a point where the two fields come together. On one hand, the activity of an AMP is commonly expressed as the threshold concentration upon which bacterial growth is inhibited (the MIC, or minimum inhibitory concentration). On the other, biophysical studies with model phospholipid membranes often identify concentration thresholds upon which the peptide behavior becomes disruptive [4]–[10]–tipically through pore formation or membrane lysis. This is an expected point of convergence between biological activity and molecular-level behavior given that the bacterial membrane has long been identified as the primary target for most AMPs; indeed, connections between in vivo MICs and thresholds in model membranes have been recently proposed [9], [11]. In this work we describe a simple physical-chemical framework that models this correlation. We then fully explore its predictive power, with good predictions for the activities of the AMPs Omiganan and BP100.

## Analysis

### Model background

Our analysis is centered on the comparison of local membrane concentrations at the threshold events of the MIC and of molecular-level membrane disruption. It therefore requires that those concentrations be known or somehow estimated.

In studies with model membranes bound concentrations can usually be directly extracted from published data when expressed as the peptide-to-lipid ratio (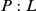) at which the threshold occurs (see the Supporting Information for involved approximations in this approach). Threshold AMP 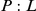 values commonly fall in the 1∶10 to 1∶100 range [5], [9], corresponding to a 13 to 130 mM range of membrane-bound peptide concentrations.

Calculating the in vivo amount of peptide molecules bound to the bacterial membrane at the MIC is, however, not as straightforward. To obtain an estimate for this value we assumed that the distribution of the peptide between the medium and the bacterial membrane obeys a simple Nernst equilibrium [12]. Under this approach, commonly used to describe binding to model membranes [3], [13], [14] and in which these are considered an immiscible lipidic phase, the partition constant 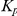 is defined as a concentration ratio:
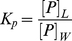
(1)where 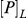 and 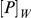 are the peptide concentration in the lipidic and aqueous phases, respectively–the Supporting Information (Text S1) details some simplifications implicit in this definition, as well as the conversion from other types of binding constants [14].

From Equation 1, the fraction of peptide molecules in the lipidic phase (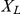) can be obtained as
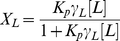
(2)where 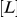 is the total lipid concentration and 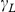 the molar volume of the lipid phase. Finally, the local peptide concentration in a membrane at a lipid concentration 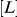 is given by
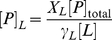
(3)where 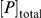 is the peptide concentration over the global volume.

Calculation of the bound peptide concentration requires that a 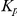 for the interaction with bacterial membranes is known. We assumed that an AMP interacts with such membranes and their model counterparts with similar affinity and, so, that binding or partition constants determined for the latter are acceptable approximations; a typical [9] AMP-membrane 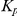 of 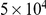 was used. Equations 2 and 3 also require knowledge of the amount of membrane lipid available for peptide binding under MIC assay conditions (thus termed 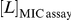). This value was estimated assuming a bacterium dry mass of 489 fg [15], 8.2% of which are phospholipids [16] (data for *E. coli*); admitting a maximum bacterial titre of 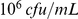 [17] this yields an 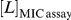 of 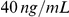, or 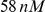 if all the phospholipids are approximated to have the molecular weight of dipalmitoylphosphatidylethanolamine (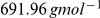). This value is in good agreement with, and corroborates, published results from distinct calculations based on bacterial surface area [18], [19] (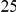 and 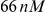, respectively).

Lastly, a 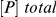 of 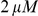 was assumed–a typical MIC value for an AMP–together with a 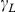 value of 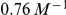, corresponding to the density of a fluid bilayer [20].

With the above parameters only 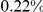 of the total peptide is predicted, by Equation 2, to bind bacterial membranes in a MIC assay. This very low fraction indicates that almost all peptide remains in the aqueous phase but it does not mean that the local concentration in the membrane is low: indeed, Equation 3, indicates a bound concentration of 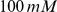. This value–about 13 phospholipids per bound peptide–falls in the range of the bound threshold concentrations in model membranes mentioned earlier, supporting the parallel between those and the MIC. The high obtained concentration also supports the proposed [9] view that, rather than being unphysiological, such high bound AMP concentrations are expectable events in vivo (indeed, even higher local concentrations in bacteria have been measured [21], although the lack of physiological ionic strength in that experiment is likely to have exacerbated the degree of binding).

### Activity prediction

The usefulness of our model was extended, in a more quantitative sense, to predict antimicrobial activities from known threshold occurrences in model membranes: Equations 2 and 3 were combined to define 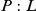 as a function of 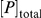, 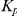 and 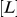:
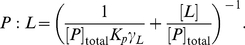
(4)Under the conditions where activity is triggered in vivo 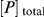 is the MIC, 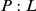 is the disruption threshold in the membrane (here termed 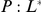) and 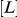 is of the magnitude of 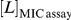:
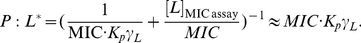
(5)The approximation in the expression is possible because the nanomolar values of 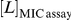 are two to three orders of magnitude smaller than the typical micromolar MICs [1], and, given average values for AMP partition constants [9], 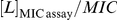 becomes negligible for the result. Finally, we arrive at the relationship between the MIC of an AMP and its disruptive behavior (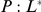) on a model membrane:
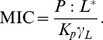
(6)At this point it should be noted that the disruption threshold 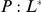 need not refer exclusively to membrane lysis or poration. If a peptide requires a given membrane concentration to translocate into the cell–even if it does so without leaking it, and subsequently only attacks inner targets–then that will still be a valid 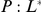 to use in Equation 6.

Equation 6 can be applied on its own to AMP threshold (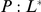) and affinity (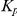) data; however, a linear relationship has been described between the global peptide and lipid concentrations in a system when threshold events occur in a membrane [7], [22]. An important feature of this relationship is that its intercept is equivalent to the MIC estimate defined in Equation 6:
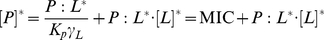
(7)where threshold-point conditions are indicated by an asterisk. Using Equation 7 MIC values can be estimated from a single experiment consisting in the determination of peptide vs. lipid threshold curves with model membranes. No explicit calculation of 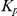 or 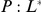 values is required–although these can be recovered if needed [7], [22]. Furthermore, because the MIC estimate only depends on the intercept of the curve, the prediction is robust to the actual lipid concentrations as long as relative dilutions between data points are kept. This avoids the need for accurate lipid quantification and introduces the possibility of using liposomes that have not been made unilamellar [23], [24] (by processes such as freeze-thaw, extrusion, or sonication), obviating a time- and resource-consuming step associated to the use of model membranes.

### Extension to hemolysis

The model was extended to predict AMP activity against red blood cells (the minimum hemolytic concentration, or MHC), which is a common measure for cytotoxicity. The only difference relative to the MIC prediction approach was the use of 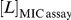 instead of 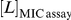. An 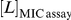 of 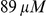 can be estimated from the concentration of erythrocytes in the human blood (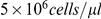 [25]), their average surface area (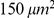 [26]), the area per phospholipid headgroup (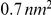 [27]), and a commonly blood dilution used in MHC determination of 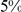 v/v [28], [29].

### Robustness of the in vivo binding model

In the calculations above an in vivo scenario was severely simplified in several aspects. It is thus important to assess the extent to which approximations affect the obtained practical and theoretical conclusions.

#### Estimation of 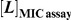


The estimation of 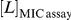 from a bacterium's weight is prone to error and implicitly assumes an average value. Likewise, the geometric estimates approximate the bacterium shape as a sphere or a simple rod, which may not be entirely accurate; the same stands for the number of leaflets–which may double if a Gram-negative outer membrane is added–and for the area per phospholipid–which will surely vary under physiological conditions. However, the precise value of 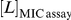 is unimportant because the term containing 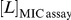 in Equation 5 is negligible when 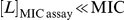, and, since (
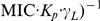
 is in the order of 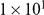, even an 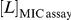 equal to the MIC will only add around 10% error to the result. As such, any errors in the approximation of 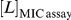 will only be of significance if they impose a correction larger than the two to three orders of magnitude by which typical MICs are greater than the estimated nanomolar lipidic concentrations.

#### Presence of other system components

This model does not explicitly take into account possible interactions of the peptide with other system components besides the cell membrane. However, for such interactions to influence the bound concentrations–namely by significantly reducing the unbound amount of peptide–they would have to be extremely strong or the interacting components would have to be in a very high concentration. One other cellular constituent present in enough quantity to potentially sequester a significant amount of peptide is the anionic Gram-positive peptidoglycan wall. Even so, this structure has at most only 20 times the volume of the membrane [30] and, despite not being the subject of many studies, a proportionally lower affinity towards it was reported for the peptide omiganan [7], meaning that the presence of peptidoglycan is roughly equivalent to having a second membrane for the peptide to interact with. This is well within the allowable error margin discussed above and it also means that the presence of an outer membrane in Gram-negative bacteria will not significantly influence the binding model.

Likewise, bacterial DNA and RNA molecules, being markedly anionic, could bind a significant portion of the peptide and render the above conclusions invalid (irrespectively of the physiological relevance of such interactions [31]). This, however, should have little impact on the results: there is a total of about 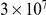 nucleotide-associated anionic charges per bacterium, taking into account average amounts of DNA, mRNA and tRNA in an E. coli cell [32], [33]. Under MIC assay conditions that number of anionic charges would bind 8 nM of a 6+ charged peptide, assuming a one-to-one charge interaction. This is 0.4% of a 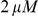 MIC–low enough not to significantly affect the estimations.

However, while cellular components seem to be unable to prevent high peptide accumulation in the membrane, the same might not be true for bulk phase constituents [18], [19], which are often present in milimolar concentrations: one can expect high ionic strengths to reduce the degree of peptide interaction with the membrane by neutralizing the effective charge of both the peptide and the membrane surface, especially if the involved counterions are not easily displaced. This effect should be compensated for by using physiological ionic strengths when measuring partition constants.

### Experimental determinaton of 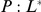


Critical 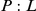 ratios were measured by adding the AMP BP100 (H-KKLFKKILKYL-NH2; synthesized as described elsewhere [34]) to suspensions of multilamellar vesicles of a 1∶2 proportion of POPC and POPG (1-palmitoyl-2-oleoyl-sn-glycero-3-phosphocholine and 1-palmitoyl-2-oleoyl-sn-glycero-3-phospho-rac-1-glycerol, from Avanti Polar Lipids, Inc.) prepared as described elsewhere [10]. Optical densities of the vesicle suspension were taken for several lipid-peptide concentration pairs using an MTX Labsystems, Inc. Multiskan EX plate reader and BD Falcon UV-transparent 96-well plates. Many of the used parameters were found through the BioNumbers database [35].

Phospholipid mixtures containing 30% POPG, or 25% POPG and 5% cardiolipin, have been growing in acceptance as accurate models of the bacterial cell membrane [36]. In this work 67% POPG were used because threshold events of BP100 were more clearly observable at higher proportions of anionic lipids. On the other hand, this proportion might actually better approximate the charge density of the Gram-negative outer membrane [37]. See the Supporting Information for an analysis of the possible impact of using this model system on the conclusions of this work.

## Results and Discussion

The predictive model was tested using Equation 6 with the published parameters and activities of the peptides omiganan [7], [38] and BP100 [10], [28], [34]. Good agreement between predicted and observed activities was obtained for both, as summarized in Table 1.

**Table 1:** Estimated and observed activities for the AMPs BP100 and omiganan against Gram-negative bacteria.

Peptide	Membrane interaction parameters^a^		MIC estimate ( 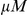 )	Observed MIC^b^ ( 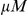 )
	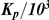	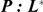		
BP100 [10], [34]	30.8–84.1	1 ∶ 8.4	1.9–5.1	2.5–5.0^c^
Omiganan [7], [38]	5.2–43.5	1 ∶ 37.0	0.8–6.8	9.0^d^

a Interaction parameters for 1∶2 POPC∶POPG systems, obtained by fluorescence spectroscopy techniques.

b Only the value/range for the most susceptible strain is indicated.

c Range corresponds to complete growth inhibition of either *Pseudomonas syringae* or *Erwinia amylovora*.

d Value corresponds to the 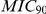 against *Escherichia coli*.

**Figure 1:**
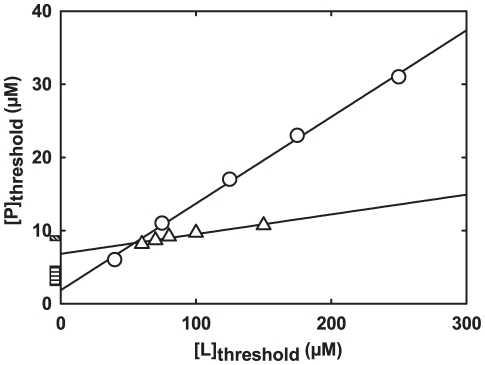
Application of the MIC prediction method to AMP-membrane threshold data. Published threshold data [7], [10] on the interaction of the AMPs BP100 (circles) and omiganan (triangles) with POPC∶POPG 1∶2 unilamellar vesicles were fit with Equation 7, yielding intercepts of 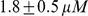 and 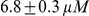, respectively. The lowest MIC values measured against Gram-negative bacteria are indicated for omiganan [38] (diagonal hatching) and BP100 [34] (horizontal hatching) next to the 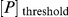 axis; the intercepts, predictive of the MIC, lie within few 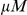 of these values.

**Figure 2:**
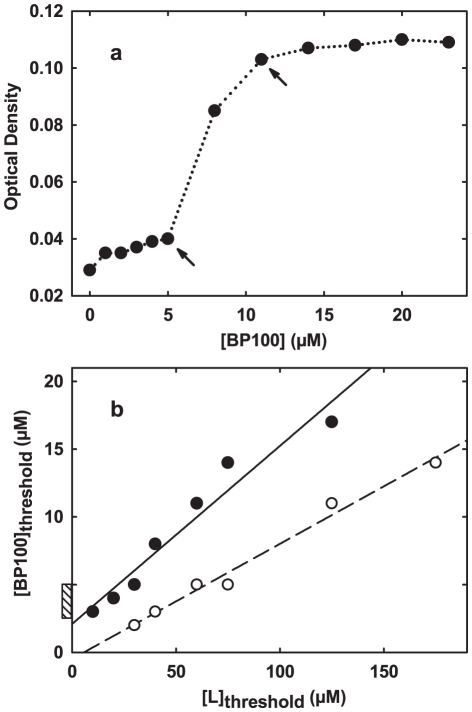
Application of the MIC prediction method to thresholds in BP100 interaction with multilamellar vesicles. a) Optical density of a suspension of multilamellar vesicles (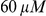 POPC∶POPG 1∶2) at different concentrations of added BP100. Arrows indicate critical threshold points. b) Plot and linear fit, according to Equation 7, of critical points in panel a) and in similar curves obtained with different lipid concentrations; empty and full symbols denote the first and second critical points, respectively, of each curve. The intercept of the line fitted to the second critical points (which correspond to the 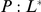 determined elsewhere [10]) estimates a MIC of 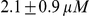. The value is both close to the estimate in Figure 1 and to the observed MIC range of BP100 [34], indicated next to the 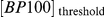 axis (hatched box).

Equation 7 was then tested with published threshold data for the same peptides, also with good approximations of the actual MICs (Figure 1). This simple approach was further tested using threshold points of BP100 interaction with multilamellar vesicles, determined from the optical density of the system. This prediction (Figure 2) is in good agreement with that from Figure 1 and the observed MICs, the method being indeed robust to the use of multilamellar vesicles.

Equations 6 and 7 may also be used to estimate other relevant limits, such as the minimum hemolytic concentration (MHC) of a peptide. The concentration of erythrocyte membrane phospholipid in an MHC assay (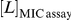) was estimated to be of almost 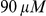; this value is of the same order of magnitude as typical MHC values of hemolytic peptides [29], [39], which is a borderline situation regarding the validity of Equation 5. The method is then more likely to estimate a lower bound of an MHC than a central value. Application of Equation 7 to published threshold data on the interaction of the AMP melittin with different erythrocyte membrane models [22], [40] predicts MHC values from 220.02 to 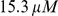. Notwithstanding the high 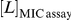 and the wide prediction interval, the values do overlap with the observed 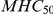 range [29], [41], between 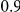 and 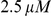.

The successful application of the method to BP100 and omiganan forebodes a good predictive power, in spite of all the simplifications and approximations in the model. Hopefully, along with an increasing awareness of the relevance of partition and threshold events to the activity of AMPs, more datasets will become available against which our method can be applied and validated.

Finally, more than a theoretical exercise in bridging biology with physical-chemistry, the presented methodology provides a basis for fast, cost-effective alternatives for screening libraries of peptide drug leads before actual biological testing. The predictive relationships can also be coupled with drug design algorithms, further improving the process. This work demonstrates that it is possible to use a purely physical-chemical reasoning to understand, model, and predict the mechanisms of complex biological interactions such as AMP-mediated bacterial death, with applications that, in this case, may ultimately lead to a faster, more efficient antibiotic drug development.

### Limitations to the application of the model

It must be remarked that although our model performed well with omiganan and BP100 it is too simple to precisely predict the activity of all AMPs against all types of bacteria. The use of the partition constant implies the assumption of equilibrium in membrane binding; this might never be attained in practical timescales for cases where bacteria present effective barriers to free diffusion towards the membrane (e. g., a very thick or cation-containing peptidoglycan layer [42]). The model can, nonetheless, account for differences in the activity of a peptide against distinct strains so long these result from differences in membrane composition, as those generally entail a change in 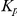 or 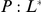.

Another limitation to the applicability of the model stems from the working hypothesis that peptide action depends on a critical membrane-bound concentration threshold: peptides like the apidaecins [43] that exert their action independently of some sort of cooperativity in the membrane are not contemplated. Still, membrane disruption by either lysis or poration is not a requirement of the model; the activity of peptides that target intracellular components can still be modelled as long as translocation into the cytoplasm is a threshold-dependent step.

Multiple disruptive thresholds are often observed with model membranes, which may complicate analysis if identification of the relevant threshold is not possible. Such is the case in Figure 2 and in one of the data sources used for predicting the MHC of melittin [22]. Lacking further information on the relationship between these disruptive points and the in vivo activity of the peptides, we opted to combine predictions from the different thresholds into a single range (as long as the predicted MIC/MHC was a positive value). This, of course, resulted in a broadened prediction interval and it is a possible reason why the MHC prediction spans almost three orders of magnitude.

Finally, predictions may be sensitive to the precise constitution of the membrane model. As stated earlier, this may justify different bacterial susceptibilities to a given AMP, but it also stresses the importance of using accurate models. An analysis of the dependence of MIC predictions on membrane anionic density has been included in the Supporting Information regarding the relatively high anionic content of the bacterial membrane model used in this work. Likewise, the lack of precision in the MHC prediction may also result from the data having been collected in three different zwitterionic erythrocyte membrane models [22], [40], two of which in the gel phase [22]. Indeed, when modelling the essentially zwitterionic erythrocyte membrane, where the dominance of electrostatic interactions is absent, one can expect peptide partition to be quite sensitive to the particular constituents used.

## Supporting Information

Text S1 Extended discussions on the **1) Analysis of published data** under the proposed model, **2) Influence of the anionic charge of the membrane models** on the conclusions of this work, **3) Approximations in the partition model**, and **4) Conversion from other constants**, obtained under different partition/binding formalisms.
(PDF)
