# Supplementary material for: Prediction of Antibacterial Activity from Physicochemical Properties of Antimicrobial Peptides
Source: PLoS One. 2011 Dec 14;6(12):e28549. doi: 10.1371/journal.pone.0028549 (PMC3237455; doi:10.1371/journal.pone.0028549)
Supplement: Text S1 — Extended discussions on the 1) Analysis of published data under the proposed model, 2) Influence of the anionic charge of the membrane models on the conclusions of this work, 3) Approximations in the partition model, and 4) Conversion from other constants, obtained under different partition/binding formalisms. (PDF) [file pone.0028549.s001.pdf]

Supporting Information for

Prediction of antibacterial activity from physicochemical properties of antimicrobial peptides

1 Analysis of published data

Published data regarding thresholds in the membrane is not always clearly presented as the bound P:L ratio. Instead, the global P:L ratio is often found. This ratio is not as accurate as the local ratio as no information is provided on the amount of peptide that is actually bound to the membrane. If K_p and the sample volume are known, a global ratio can be corrected, but if no binding information is available approximations must be made: a shortcut to obtaining the local ratio without exact knowledge of K_p is to consider X_L very close to 1, in which case the global P:L ratio will tend to the value of the local ratio. From Eq. (2) in the main article it can be seen that this is valid if $[L]$ is large enough so that $K_p \gamma_L [L]$ is much greater than 1. For a K_p of 5×10^4 more than 90% of bound peptide will be attained for $[L]$ greater than 0.3 mM. Because many studies with AMPs are in such conditions, one can usually approximate the global P:L ratio as the local one, if no better alternatives are available.

2 Influence of the anionic charge of the membrane models

As stated in the main text, the use of a 2:1 POPG:POPC membrane system, instead of a system with roughly half the anionic charge density, may depart form physiological relevance. It is therefore important to assess how the increased anionicity may impact the conclusions in this work.

The MIC prediction will essentially depend on how the the Kp and threshold ratio are affected by an increased membrane anionic charge. From data on BP100 [1] it can be seen that although the Kp value increases with the anionic content of the membrane it remains in the 10^4 order of magnitude. On the other hand, thresholds involving charge events (such as full or partial charge neutralization) can be expected to vary proportionally to the increase in anionic content–corresponding in this case to a twofold increase. These variations in K_p and P:L^* are not only small (to an order of magnitude) but, more importantly, will partly cancel out in Eq. (6) in the main article, suggesting that the prediction is robust to the precise anionic content of the membrane model.

3 Approximations in the partition model

There are two simplifications implicit in the definition of the partition constant and partitioned peptide fraction (Eqs. (1) and (2) in the main article) where it is assumed that the amounts of peptide in the membrane ($n_{p,L}$) and in the aqueous phase ($n_{p,W}$) are small compared to the total amount of lipid (n_L) and water (n_W) molecules, respectively [2]. While $n_{p,W} \ll n_W$ is valid for practical peptide concentrations, $n_{p,L} \ll n_L$ may not always be true for some of the high concentrations considered. The bound peptide concentration can be corrected by using the full K_p definition [2]:

$$K_p = \frac{\dfrac{n_{p,L}}{(n_L + n_{p,L})\gamma_L}}{\dfrac{n_{p,W}}{(n_W + n_{p,W})\gamma_W}} \approx \frac{\dfrac{[P]_L}{1+\gamma_L [P]_L}}{[P]_W} \Leftrightarrow$$

$$\Leftrightarrow [P]_L = \frac{K_p [P]_W}{1 - \gamma_L K_p [P]_W} \quad (1)$$

where γ_L and γ_W are, respectively, the molar volumes of the lipid and water molecules; the approximation in the expression can be made after assuming $n_{p,W} \ll n_W$. Using Eqs. (1) (of the main article) and (1), the relative change in the $[P]_L$ value upon correction can be obtained (the 'c' subscript denotes a corrected parameter):

$$\frac{[P]_{L,c}}{[P]_L} = \frac{\dfrac{K_{p,c} \cdot [P]_{W,c}}{1 - \gamma_L K_{p,c} \cdot [P]_{W,c}}}{K_p \cdot [P]_W} \quad (2)$$

One can assume that K_p values, being calculated away from excessively high peptide densities in the membrane, are equivalent to $K_{p,c}$. In addition, admitting that $[P]_W$ will not change significantly in vivo (see main text), $[P]_{W,c}$ can be made approximately equal to $[P]_W$. Thus,

$$\frac{[P]_{L,c}}{[P]_L} = \frac{1}{1 - \gamma_L K_p [P]_W} = \frac{1}{1 - \gamma_L [P]_L} = \frac{1}{1 - P{:}L} \quad (3)$$

This correction, even at $[P]_L = 130$mM (P:L = 1:10), amounts to a difference of only 11% in the corrected concentration. Furthermore, it is a correction in the direction of higher bound concentrations. Rather than invalidating any of the conclusions in the analysis this further approximates the results to saturation.

It should be borne in mind that, besides the above correction, the entire partition formalism as presented here is based on the assumption of the two phases being ideally diluted solutions (i.e. the peptide molecules are dilute enough not to significantly interact with each other). As one approaches high extents of membrane coverage this assumption is inevitably invalidated. However, the fact that peptide vs. lipid fluorescence curves for BP100 and omiganan are essentially linear up to membrane saturation [1, 3] suggests that there is little error introduced by having high bound concentrations, in that range of conditions. This means that the partition equilibrium approach is valid to calculate bound concentrations at threshold points that occur below, or at, the membrane saturation point.

4 Conversion from other constants

A different binding constant, K_b (M^{-1})–or its inverse, the dissociation constant K_d (M)–is also commonly used, where it is assumed that the peptide interacts with the membrane phospholipids to form a 1:1 complex [4] with reaction equilibrium constant K_b; the validity of this approach to interpret membrane partitioning is discussed elsewhere [2, 5]. Because published data are sometimes analyzed in this alternative framework, a conversion between both K_p and K_b is presented. K_b is defined as:

$$K_b = \frac{[PL_{\text{complex}}]}{[P]_{\text{free}}[L]_{\text{free}}} \approx \frac{X_L[P]}{(1 - X_L)[P][L]} = \frac{X_L}{(1 - X_L)[L]} \quad (4)$$

where $[PL_{\text{complex}}]$ is the concentration of the 1:1 peptide:lipid complex, $[P]_{\text{free}}$, $[P]$, $[L]_{\text{free}}$ and $[L]$ are the unbound and the total concentrations of peptide and phospholipid, respectively, and X_L is the mole fraction of the bound peptide. The approximation of $[L]_{\text{free}}$ to $[L]$ significantly simplifies the calculations, but, in the case of high extents of binding, an error will be introduced. Because this approximation is roughly equivalent to the assumption of $n_{p,L} \ll n_L$ in the previous section, the resulting correction of the bound concentrations will be subject to an error of similar magnitude. Eq. (4) can be solved for X_L:

$$X_L = \frac{K_b[L]}{1 + K_b[L]} \quad (5)$$

It then becomes obvious, by comparison with Eq. (2) in the main article, that in the limit of weak interactions:

$$K_p = K_b/\gamma_L \quad (6)$$

References

[1] Ferre R, Melo MN, Correia AD, Feliu L, Bardají E, et al. (2009) Synergistic effects of the membrane actions of cecropin-melittin antimicrobial hybrid peptide BP100. Biophys J 96: 1815–1827.

[2] Santos NC, Prieto M, Castanho MA (2003) Quantifying molecular partition into model systems of biomembranes: an emphasis on optical spectroscopic methods. Biochim Biophys Acta 1612: 123–135.

[3] Melo MN, Castanho MA (2007) Omiganan interaction with bacterial membranes and cell wall models. Assigning a biological role to saturation. Biochim Biophys Acta 1768: 1277–1290.

[4] Christiaens B, Symoens S, Verheyden S, Engelborghs Y, Joliot A, et al. (2002) Tryptophan fluorescence study of the interaction of penetratin peptides with model membranes. Eur J Biochem 269: 2918–2926.

[5] Matos C, Lima JL, Reis S, Lopes A, Bastos M (2004) Interaction of antiinflammatory drugs with EPC liposomes: calorimetric study in a broad concentration range. Biophys J 86: 946–954.
